# Assessment of the Analgesic Effects of Extrapleural Infusion of Ropivacaine in Neonates with Esophageal Atresia (EA) Repair

**Published:** 2010

**Authors:** Mohsen Rouzrokh, Alireza Mirkheshti, Alireza Mirshemirani, Afsaneh Sadeghi, Azita Tavassoli, Ahmad Khaleghnejad Tabari

**Affiliations:** a ***Pediatric Surgery Research Center, Mofid Children’s Hospital, Shaheed Beheshti University of Medical Sciences, Tehran, Iran.***; b ***Mofid Children’s Hospital, Shaheed Beheshti University of Medical Sciences, Tehran, Iran.***; c ***Aliasghar Children’s Hospital, Iran University of Medical Sciences, Tehran, Iran. ***

**Keywords:** Pain, Infant, Extrapleural, Ropivacaine, Esophageal atresia

## Abstract

Insufficient control of post-thoracotomy pain can produce breathing dysfunction and long term staying in neonatal intensive care unit (NICU). It can increase the incidence of pulmonary complications such as atelectasis, pneumonia and respiratory failure. The aim of this study was to determine the analgesic effect of continuous extrapleural nerve block, using ropivacaine, in neonates younger than 7 days old with esophageal atersia (EA) and the incidence of atelectasis and duration of hospitalization in NICU.

For this purpose, from February 2007 till January 2009 in Mofid children’s hospital, 68 neonates under 7 days old whom were candidate for thoracotomy due to esophageal atresia were, randomly divided into two groups in a controlled clinical trial. The cases received extrapleural infusion of ropivacaine 0.5% (0.1 mL/kg/h for 48 h) and controls received acetaminophen 20 mg/kg three times a day via the rectal route. Hemodynamically unstable patients and those who suffered from hospital infections were excluded from the study. After the surgery, all patients had spontaneous breathing without endotracheal tube and stable hemodynamic in NICU. Pain level was determined for each neonate, based on the neonatal infant pain scale (NIPS) grading. The incidence of atelectasis in the first 48 h after operation and throughout the NICU staying were also determined.

Results showed that there were no significant difference in the mean age, sex proportions and mean weight between the two groups. The mean pain score in the group received ropivacaine (1.9 ± 0.7) was significantly less than the control group (5.2 ± 0.6) (p < 0.001).

Five percent of cases (n = 1) and 100% of the control group (n=20) had pain scores equal or greater than 3 (p < 0.001). The incidence of atelectasis among cases was less than the control group (35% vs. 65% respectively; p = 0.58). Duration of hospitalization in the case group (12 ± 5.6 days) had no significant difference from the control group (13.6 ± 4.8 days) (p = 0.3)

In conclusion, the results showed that continuous extrapleural infusion of ropivacaine reduces the pain noticeably and atelectasis relatively, after thoracotomy in neonates younger than 7 days suffering from EA, compared to the acetaminophen group.

## Introduction

Pain is an unpleasant physical or spiritual sense due to stimulation of specific nerve endings. Pain threshold in infants is low and there is a negative relationship between pain threshold and age (1). Incomplete control of pain after surgery can produce superficial breathing, chest immobility and decrease in the force of coughing (2-4).

Moreover, long term hospitalization may have a role in increasing the incidence and severity of pulmonary complication, such as pneumonia or respiratory failure by incomplete control of pain (2). 

Systemic procedures for pain control have significant side effects and little efficacy in pain control (5). Ropivacaine is a long acting local anesthetic, with only a few side effects. By insertion of an extrapleural catheter near the intercostal nerves under direct vision, continuous infusion of local anesthetic was performed by this technique, and effective pain control after thoracotomy was found to be equal to other systemic and epidural procedures. Moreover, sympathetic plexus and systemic hypotension avoided by this technique was comparable to the epidural technique (4). Respiratory depression and sedative state of systemic opioids have not been seen by the extrapleural technique. Hence, there would be little need for careful monitoring of patients. 

In this survey, we compared the analgesic effect of extrapleural infusion of ropivacaine to rectal acetaminophen in less than 7 days old neonates after esophageal atresia surgeries, in Mofid children’s hospital, as the primary outcome and studied the incidence of atelectasis and duration of hospitalization in NICU as the secondary outcome. 

## Experimental

In order to evaluate the efficacy of analgesic properties and incidence of atelectasis after EA and duration of NICU staying in less than 7 days old neonates, a randomized controlled trial was preformed in Mofid children hospital from February 2007 till January 2009. In this survey, 68 neonates younger than 7 days, whom were candidate for EA repair, selected and after confirmation by the research committee and obtaining written consents from the neonates’ parents about participation in the survey, the patients were randomly divided into two groups. This study was then conducted as a controlled clinical trial, with 34 patients placed in the case group and 34 patients in the control group. All neonates had esophageal atresia and thoracotomy. Furthermore, all the neonates with major congenital heart disease were excluded from this study. The patients in case group received extrapleural infusion of ropivacaine (0.1 mL/kg/h) for 48 h and the control group received acetaminophen (20 mg/kg) three times a day rectally, for the same period of time. Hemodynamically unstable patients and those who suffered from hospital infection were excluded from the study. In the data form of every neonate, according to patient’s evidence and paraclinical laboratory tests, all the information regarding the neonate’s age (days), sex, pain score (according to NIPS) and atelectasis during 48 h after surgery and duration of NICU stay were noted. All patients had spontaneous breathing after operation. In the case group, after performing the operation and while the neonates were anesthetized, a feeding tube (number 5, made by Maersk Medical Company) inserted by the surgeon into the extrapleural space, next to pleura and intercostal nerves. Then, 10 mL vials of ropivacaine (Naropin, made by Astra Zeneca Company) were diluted by normal saline to a concentration of 0.5%, and infused as 0.1 mL/kg/h for 48 h. The pain scale was used based on the NIPS score, and contained parameters of crying, facial expression, breathing patterns, legs and arms movements and state of arousal (6).

Based on this system, scoring would be from zero to seven. The score which is equal or greater than three would be significant for pain. During 48 h, and at 4 h intervals, patients’ pain score was evaluated by one of the three trained head nurses of NICU, and the scores were recorded in a data from. The mean of 13 times of scoring was noted as the “average pain score”. We also studied the results of atelectasis and NICU hospitalization in both groups. All neonates whom were candidate for surgery had chest X-ray before and after operation. The diagnosis of atelectasis was based on the presence of fever and chest X-ray findings reported by a fixed radiologist. In febrile neonates, primary opacities in chest X-ray were due to aspiration and new opacities were because of atelectasis. 

All the data gathered were analysed by the SPSS 12 statistical software. For the purpose of comparison, data were examined statistically using the Chi-square test as well as the student’s t-test. The p-values less than 0.05 were considered as significant. 

## Results

There was no significant difference (p > 0.05) between the two groups in terms of the mean age, sex and weight proportions. In the case group, the average age was 2.6 ± 0.6 days and in the control group the average age was 2.6 ± 0.7. In the case group, 18 patients were male and 16 were female and in the control group, 17 were male and 17 female. In the case group the mean weight was 3834 ± 359 g and in the control group it was 2715 ± 637 g. There was no significant difference between the weight of both groups (p < 0.05). 

The mean pain score in the group received ropivacaine (1.91 ± 0.7) was significantly (p < 0.05) less than the control group (5.53 ± 0.72).

In our analysis, the mean pain score in the patients whom received ropivacaine was significantly less than the patients which had rectal acetaminophen (p < 0.001). In terms of the description of pain, based on the pain score (Table 1), the number of patients who had a pain score equal or more than 3, was only one (2.9%), but in the control group, all infants (n = 34) had pain scores equal or greater than 3 (p < 0.001). Atelectasis in the case group was 32.35% (11 patients) and in the control group was 67.64% (28 patients) as could be seen in Table 2. 

**Table 1:** The mean pain score in the case and control groups

**Groups**	**Number of patients**	**Mean ± SD**
Case	34	1.91 ± 0.7
Control	34	5.53 ± 0.72

p-value < 0.0001

**Table 2:** Atelectasis in the case and control groups

	**Negative**	**Positive**	**Total**
Case	23	11	34
Control	11	23	34
Total	34	34	68

The chi-square test showed that the atelectasis proportion in the case group was significantly different from control group (X2: 7.11, DF=1, p = 0.007). Duration of hospitalization in NICU of the case group was 12 ± 5.6 days and in the control group was 13.6 ± 4.8 days. The statistical analysis showed that there was no significant difference in the duration of hospitalization between the two groups (p > 0.05). 

## Discussion

By performing our survey, we concluded that analgesia by extrapleural ropivacaine after EA operation in neonates has considerable effects compared to rectal administration of acetaminophen. Moreover, extrapleural ropivacaine, compared to rectal acetaminophen, resulted in a relative decrease in the incidence of atelectasis. By matching some factors such as age, sex and weight in our survey, it was found that the effects of these variables had been omitted well, and since the only obvious difference between the two groups was due to the type of analgesia, it is predicted that differences were due to the effect of extrapleural ropivacaine infusion. 

In the Meyer’s survey, pain scores and the amount of opioids consumed, revealed that pain was controlled adequately after surgery (6). In the Marret’s survey, the average VAS score during resting and coughing was significantly low in the thoracic paravertebral block (TPVB) group (7). In our survey, the mean pain score in the case group was significantly less than that of the control group. In the Tetik’s survey, atelectasis and pneumonia after operation were observed in 4 and 2 patients, subsequently within the saline group. However, there were no such side effects in the bupivacaine group (8). Considering the differences between bupivacaine and ropivacaine, we also found the same results, such that atelectasis in our case group was significantly less than that of the control group. 

Marret studied on paravertebral ropivacaine, but Meyer and our survey focused on intercostal extrapleural blocks (7). Randa and Badawi studied on total paravertebral block (TPVB) by ropivacaine alone and compared it to the mixed intrapleural ketamine-ropivacaine and concluded that TPVB by ropivacaine alone, compared to interapleural ketamine-ropivacaine, is superior in pain control after thoracoplasty in scoliosis surgeries (9). TPVB has lower VAS scores during walking and coughing and a lower need to morphine and better pulmonary function test. In this study, we can again see the appropriate effect and low complication of ropivacaine, which are similar to our findings. 

It should be stated that we did not assess the need for additional analgesia, as an indicator of effective analgesia. However, pain scores in the case group certified effective analgesia after thoracotomy. Another point in our survey is the similarity between the duration of hospitalization in both groups, which in contrast to previous studies, especially on bupivacaine. It should be said that a number of interfering agents play role in hospitalization, such as antibiotic therapy, type of NICU protocols and associated anomalies. Therefore, we could not exactly detect the role of ropivacaine in the duration of hospitalization, and expect that through further studies, the confining bias would be omitted. Furthermore, the need for additional analgesia should be studied. 

Finally, we think that the most important limitation in our survey was awareness of the nurses who evaluated pain scores and unfortunately, we had no way to resolve this problem. 

In conclusion, it could be said that extrapleural infusion of ropivacaine after esophageal atresia repair in neonates is more appropriate for decreasing the level of pain and atelectasis than rectal administration of acetaminophen. Even though we did not encounter any side effects using this technique, however, it is proposed that further researches should be carried out in order to confirm the results of our survey. 
